# Mechanisms of Inflammation in Neutrophil-Mediated Skin Diseases

**DOI:** 10.3389/fimmu.2019.01059

**Published:** 2019-05-08

**Authors:** Angelo V. Marzano, Alex G. Ortega-Loayza, Michael Heath, Daniel Morse, Giovanni Genovese, Massimo Cugno

**Affiliations:** ^1^UOC Dermatologia, Fondazione IRCCS Ca' Granda Ospedale Maggiore Policlinico, Milan, Italy; ^2^Dipartimento di Fisiopatologia Medico-Chirurgica e dei Trapianti, Università degli Studi di Milano, Milan, Italy; ^3^Department of Dermatology, OHSU Wound Care and Hyperbaric Medicine, Oregon Health and Science University, Portland, OR, United States; ^4^Department of Dermatology, Oregon Health and Science University, Portland, OR, United States; ^5^Medicina Interna, Fondazione IRCCS Ca' Granda Ospedale Maggiore Policlinico, Milan, Italy

**Keywords:** neutrophil-mediated skin diseases, autoinflammation, inflammasome, cytokines, pyoderma gangrenosum

## Abstract

Neutrophil-mediated skin diseases, originally named neutrophilic dermatoses (NDs), are a group of conditions due to an altered neutrophil recruitment and activation, characterized by polymorphic cutaneous manifestations with possible internal organ involvement. Although a number of diseases are included in this setting, the two prototypic forms are pyoderma gangrenosum (PG) and Sweet's syndrome (SS) which usually present with skin ulcers and plaque-type lesions, respectively. They have central features significantly overlapping with autoinflammatory conditions which manifest as repeated episodes of tissue inflammation. However, in contrast to appropriate inflammatory responses to insults or to autoimmune disease, there is an absence of identifiable pathogens, autoantibodies, or autoreactive lymphocytes. The recognition of monogenic autoinflammatory diseases which can present with NDs has led to study several genes involved in autoinflammation in NDs. Based on discovering of a number of mutations involving different autoinflammatory genes, neutrophil-mediated skin diseases are nowadays regarded as a spectrum of polygenic autoinflammatory conditions. Although disease mechanisms have not yet been completely elucidated, NDs are recognized as diseases involving dysfunctional cellular signaling mediated by pathways mainly related to inflammasome and IL-1 with the contributory role of IL-17 and other effector molecules. The precise elucidation of the above-mentioned pathologic mechanisms may pave the way to tailored treatments for patients with different neutrophil-mediated skin diseases.

## Introduction

Neutrophilic dermatoses (NDs) are a heterogenous subset of conditions with common features and overlapping pathophysiology. They primarily present with cutaneous manifestations due to accumulation of neutrophils but may affect additional tissues. The most well-defined NDs include pyoderma gangrenosum (PG), Sweet's syndrome (SS), subcorneal pustular dermatosis, neutrophilic eccrine hidradenitis (NEH), bowel-associated dermatosis-arthritis syndrome (BADAS), rheumatoid neutrophilic dermatitis, Behçet's disease (BD), amicrobial pustulosis of the folds (APF) and generalized pustular psoriasis. The presentations by pathology of the neutrophil-mediated autoinflammatory skin diseases for which there is genetic and immunological evidence are reported in Table 1. As PG with its syndromic forms is the prototypical ND and has a large body of research available, it will be the focal point of the discussion. Concerning the other entities, since there are no extensive research data available in the literature, their links with suggested mechanisms would be speculative. Each disease may present within an overlapping spectrum both clinically and histopathologically which can make diagnosis difficult and management challenging. The central features of NDs have significant overlap with disorders included within the spectrum of autoinflammatory conditions which manifest as reoccurring periods of tissue inflammation (18). However, in contrast to appropriate inflammatory responses to insults or to autoimmune disease, there is an absence of identifiable pathogens, autoantibodies, or autoreactive lymphocytes (16). The concept of autoinflammation arose out of the discovery of conditions resulting from specific genetic mutations leading to chronic inflammation devoid of autoreactive T cells (or antigen specific T cells) or autoantibodies. The first autoinflammatory disease identified, TNF (tumor necrosis factor) receptor-associated periodic syndrome (TRAPS), was described in 1999 upon identification of TNF receptor mutations in the autosomal dominant condition (19). Dysregulation of innate immunity signaling pathways particularly the overexpression of the proinflammatory cytokine interleukin (IL)-1, is considered to be the prominent mechanism behind the pathophysiology of these disorders (20).

**Table 1:** Clinical, genetic, and immunological features of the main autoinflammatory neutrophil-mediated skin diseases.

**Disease**	**Cutaneous presentation**	**Genetics**	**Immunology**	**References**
PG	Ulcers with undermined, erythematous-violaceous borders	MEFV, NLRP3, NLRP12, NOD2, and LPIN2 mutations	Increased skin IL1β, IL1RI, IL1RII, TNFβ, TNFRI, TNFRII, IL17, IL17R, L-selectin, IL8, CXCL 1/2/3, CXCL16, RANTES, MMP-2, MMP-9, TIMP-1, TIMP-2, Siglec 5, Siglec 9, Fas, FasL, CD40, and CD40L	(1, 2)
		R52Q mutation in the PSTPIP1 gene	Not evaluated	(3)
		G258A and R52Q mutations in the PSTPIP1 gene	Not evaluated	(4)
		Not evaluated	Increased skin IL23	(5)
		Ptpn6 mutations	Increased serum IL1α	(6–8)
PAPA	Ulcers with undermined, erythematous-violaceous borders; inflammatory acne	E250 K in the PSTPIP1 gene	Increased serum IL1β	(9)
		E250Q and A230T mutations in the PSTPIP1 gene	Not evaluated	(10)
PASH	Ulcers with undermined, erythematous-violaceous borders; nodules, abscesses, fistulae	p.I591T, p.M694 V, p.V726A mutations in the MEFV gene; p.R702 W and p.G908R in the NOD2 gene; p.Q703 K in the NLRP3 gene; p.A106T in the IL1RN gene; p.E277D in the PSTPIP1 gene; and p.G8R in the PSMB8 gene	Increased skin IL1β, IL1RI, IL1RII, TNFα, TNFRI, TNFRII, IL-17, IL17R, L-selectin, IL-8, CXCL1/2/3, CXCL16, RANTES, MMP-2, MMP-9, TIMP-1, TIMP-2, Siglec 5, Siglec 9, Fas, FasL, CD40, and CD40L	(1)
		Increased CCTG microsatellite repeats in the PSTPIP1 gene	Not evaluated	(11)
		MEFV, NLRP3, NLRP12, NOD2, and LPIN2 mutations	IL-1-b, IL-17, TNFα, IL-8, CXCL1/2/3, and CXCL16	(2)
PAPASH	Ulcers with undermined, erythematous-violaceous borders; nodules, abscesses, fistulae	p.E277D missense mutation in the PSTPIP1 gene	Not evaluated	(12)
DIRA	Generalized pustular psoriasis	Monogenic (IL1RN mutations)	Increased serum IL1α, macrophage inflammatory protein 1α, TNFα, IL8, and IL6	(13)
DITRA	Generalized pustular psoriasis	Monogenic (IL36RN mutations)	Increased keratinocyte production of IL8 in response to proinflammatory cytokines (IL36α, IL36β, and IL36γ) as well as to IL1β and polyinosinic–polycytidylic acid	(14, 15)
CAPS	Urticaria-like lesions	Monogenic (NLRP3 mutations)	Increased serum IL1β and IL18	(16, 17)

*CAPS, Cryopyrin-Associated Periodic Syndromes; CD40, cluster of differentiation 40; CD40, CD40 ligand; CXCL, chemokine (C-X-C motif) ligand; DIRA, Deficiency of IL-1 receptor antagonist; DITRA, Deficiency of IL-36 receptor antagonist; E-selectin, endothelial selectin; IL, interleukin; IL1RN, interleukin-1 receptor antagonist; IL-xR, interleukin-x receptor; LPIN2, Lipin 2; L-selectin, leukocyte selectin; MEFV, Mediterranean fever; MMP, matrix metalloproteinase; NLRP, nucleotide-binding domain, leucine-rich repeat containing gene family, pyrin domain-containing protein; NOD2, Nucleotide-binding oligomerization domain-containing protein 2; PAPA, Pyogenic sterile arthritis, Pyoderma Gangrenosum, and acne; PAPASH, Pyogenic sterile arthritis, PG, and acne; PASH, Pyoderma Gangrenosum, Acne and Suppurative Hidradenitis; PG, Pyoderma Gangrenosum; PSMB8, proteasome subunit beta 8; PSTPIP1, proline–serine–threonine phosphatase interactive protein 1; RANTES, Regulated upon Activation, Normal T cell Expressed, and Secreted; Siglec, Sialic acid-binding immunoglobulin-type lectins; TIMP, Tissue inhibitor of metalloproteinases; TNF, tumor necrosis factor; TNFR, tumor necrosis factor receptor; VEGF, vascular endothelial growth factor*.

## Mechanisms of Inflammation in Neutrophilic Dermatoses

### Insights From Inherited Autoinflammatory Syndromes

The recognition of several monogenic diseases which can present with ND has led to an improved understanding of the possible mechanisms of polygenic non-mendelian inherited ND. These monogenic syndromes include CAPS (Cryopyrin-Associated Periodic Syndromes), DIRA [Deficiency of IL-1 receptor antagonist (IL-1RA)], DITRA [Deficiency of IL-36 receptor antagonist (IL-36RA)], PAPA (Pyogenic sterile arthritis, PG, and acne), and chronic recurrent multifocal osteomyelitis (CRMO) (21, 22).

CAPS are a group of rare inherited inflammatory disorders associated with dominant mutations in the cryopyrin-coding gene NLRP3 (nucleotide-binding domain, leucine-rich repeat containing gene family, pyrin domain-containing protein 3) on chromosome 1q44 which is also known as CIAS1, PYPAF1, or NALP3. Currently, more than 90 mutations involving NLRP3 and associated with CAPS phenotypes have been reported.

CAPS contain a spectrum of hereditary periodic fever syndromes including familial cold autoinflammatory syndrome (FCAS), Muckle-Wells Syndrome (MWS), and chronic infantile neurological cutaneous and articular syndrome (CINCA), also known as neonatal-onset multisystem inflammatory disease (NOMID). Characteristic symptoms are periodic fever and urticarial lesions. Dependent on severity, they can be associated with several clinical manifestations, including arthritis, conjunctivitis, amyloidosis, sensorineural hearing loss, aseptic meningitis, and/or cerebral atrophy (17).

DIRA is an autosomal recessive mutation in IL1RN (IL-1RA gene) on chromosome 2 leading to the absence of IL-1RA, resulting in an IL-1 signaling hyperactivity (13, 23). DIRA manifest as perinatal-onset pustular dermatitis, joint swelling, painful osteolytic lesions, and periostitis.

DITRA is caused by homozygous or compound heterozygous damaging mutations in IL36RN and is characterized by generalized pustular rashes and systemic inflammation (14, 15). IL36RN encodes for IL-36RA which inhibits binding of IL-36 to its receptor. When IL36RA is functionally impaired there is an enhanced IL-36R signaling which directly and indirectly attracts immune cells, especially neutrophils, giving rise to the pustular rashes (14, 15). IL-36 has been reported to be upregulated in a psoriasis-like inflammatory mouse model (24), confirming the role of this cytokine family in the pathogenesis of pustular psoriasis (25). Moreover, a strong correlation has been demonstrated in human psoriatic skin between the expression of IL-36 and that of other cytokines, such as IL-17, IL-23, TNF-α, and IFN-γ (26), suggesting that a positive gene expression loop might occur in psoriasis. Moreover, IL-36 also enhances IL-1α levels, further amplifying the inflammatory network (27). Thus, the IL-1/IL-36 inflammatory axis appears to be a key player of disease pathology in generalized pustular psoriasis (28) and its role may be intriguingly hypothesized also in other NDs, although it needs to be confirmed by dedicated studies.

PAPA syndrome is due to two primary mutations (A230T and E250Q) in the gene encoding proline–serine–threonine phosphatase interactive protein 1 (PSTPIP1) (10, 29). Hyperphosphorylation of the mutated PSTPIP1 protein results in increased pyrin mediated activation of the inflammasome, dysregulation of caspase 1, and overexpression of IL-1β (29). The presence of a prototypical ND such as PG in the context of PAPA syndrome, caused by a single inflammasome regulator gene mutation, suggests an autoinflammatory component in the pathophysiology of NDs (30). The occurrence of mutations in a single gene encoding an inflammasome regulating protein, as seen in PAPA, is similar to what happens in the first monogenic autoinflammatory conditions identified, i.e., familial Mediterranean fever and CAPS. Yet further studies have identified autoinflammatory syndromes with features of ND that are linked to mutations in multiple genes. In addition to PAPA, PG can present with other autoinflammatory syndromes including PG, acne, and suppurative hidradenitis (PASH) and pyogenic arthritis, PG, acne, and suppurative hidradenitis (PAPASH). The findings of these syndromic forms of PG along with reports of familial presentations of PG suggest the shared genetic basis of this ND (1, 11, 12, 31, 32).

Investigation of 7 cases of PASH and 13 cases of isolated PG revealed multiple mutations in a variety of autoinflammatory genes, including PSTPIP1, Mediterranean fever (MEFV), Nucleotide-binding oligomerization domain-containing protein 2 (NOD2), NLRP3, NLRP12, Lipin 2 (LPIN2) (2). The MEFV gene encodes for the protein pyrin, which is an innate immune system sensor which plays a central role in inflammasome activation. Different mutations in the genetic sequence can lead to variable clinical presentations with overlapping features.

In NDs, MEFV (S242R) mutations have been identified and lead to a chronically active pathogen-related response with inflammasome activation and IL-1β secretion (33). NOD2 is an intracellular pattern recognition receptors (PRRs) that plays a key role in orchestrating the proper assembly of autophagy-related proteins. Autophagy involves a cellular response resulting in the degradation of cytoplasmic components and is important in the transfer of microbial components to intracellular PRRs. The catabolic autophagy pathway responds to a wide variety of cellular stressors including nutrient deprivation, hypoxia, DNA damage, mechanical injury, reactive oxygen species (ROS), and the presence of microbial ligands (34). Loss of function mutations in NOD2 result in impaired autophagy and have been associated with inflammatory bowel disease (IBD), a condition characterized by heightened production of proinflammatory cytokines and often associated with ND comorbidity (35). Autophagy has been shown to be important in mitochondrial homeostasis (36) and its deficiency is associated with increased mitochondrial membrane permeability and ROS production, and the release of mitochondrial DNA into the cytosol (36–38). Mitochondrial DNA and ROS are both activators of the NLRP3 inflammasome (36, 37). Studies have revealed that macrophages with defective autophagy have decreased NLRP3 inflammasome activation (38). We speculate that mutations in NOD2 lead to defective autophagy causing increased production of ROS and the release of mitochondrial DNA resulting in NLRP3 activation and subsequent ND. These findings point to the polygenic basis of NDs and helped establish the categorization of NDs among the autoinflammatory disorders. The polygenic nature suggests that NDs arise from a multifactorial response in genetically predisposed patients.

### Neutrophil Production and Recruitment

In normal physiology, neutrophils are heralded as major effector cells in acute inflammation. As the most abundant leukocytes in circulation, neutrophils have been extensively described as protagonists against infection and innate immune system responders to insults. Within the bone marrow, the production and differentiation of neutrophils is regulated primarily by granulocyte colony-stimulating factor (G-CSF) (39). G-CSF also acts to promote release of mature neutrophils from the bone marrow into circulation. This is accomplished by the uncoupling of the CXC-chemokine receptor 4 (CXCR4) and CXC-chemokine ligand 12 (CXCL12) (39).

Within tissues, the “neutrostat” loop is a feedback pathway which normally suppresses neutrophilic response by suppressing production of G-CSF. This loop is initiated after phagocytosis of infiltrative neutrophils, resulting in suppression of IL-23 production by resident macrophages and dendritic cells and subsequent decreased secretion of IL-17, an important promoter of G-CSF production (40–42). Evaluation of patients with NDs has shown significantly elevated serum G-CSF, and therapeutic G-CSF is implicated in a majority of drug-induced SS cases, indicating a plausible but undiscovered contributing mechanism underlying ND (43–47). In addition, elevated IL-17 levels have been found in tissue samples from patients with PG, SS, and APF, a rare ND presenting with pustular lesions typically involving the skin folds and anogenital area (48–52). IL-17 is a key cytokine in both activation and induction of neutrophils to produce IL-8, a potent chemokine that is the principle chemoattractant of neutrophils. Increased levels of IL-8 have been found in ND lesional skin, and the chemokine works synergistically with TNF-α to potentiate and maintain a proinflammatory state (1, 2, 16, 18–21, 49, 50, 53). In mice models, experiments have revealed that protein kinase C α (PKCα) within keratinocytes promotes neutrophil infiltration of the epidermis, and may also play a central role in upregulation of G-CSF and IL-6 gene expression independent of TNF-α signaling, giving the possibility of a peripheral mechanism of ND (54).

### Innate Immune System Activation

The innate immune system uses germline encoded PRRs to recognize pathogen-associated molecular patterns (PAMPs), including (foreign) PAMPs and (endogenous) damage-associated molecular patterns (DAMPs), to initiate the production of proinflammatory cytokines (55). Changes in local cellular homeostasis including temperature, pH, oxygen, and osmolarity are recognized as DAMPs by resident tissue macrophages (55). Recognition of DAMPs initiates an inflammatory cascade involving the inflammasome which consists of a central scaffold of proteins, a sensor (including Nod-like Receptors), the adaptor protein ASC [apoptosis associated speck-like protein containing a caspase-associated recruitment domain (CARD)] and the effector protein caspase-1 (22). Members of the NLRP (Nucleotide-binding oligomerization domain, Leucine-rich Repeat and Pyrin domain-containing) family, are the primary cytoplasmic PRRs that mediate inflammasome activation (55). Oligomerization of the inflammasome results in caspase-1 activation leading to the cleavage of pro-IL-1β and pro-IL-18 to IL-1β and IL-18 (22, 55). Gain-of-function mutations in NLRP3 gene are responsible for the development of autosomal dominant inflammatory disorders which typically presents with episodic urticarial neutrophil-rich cutaneous lesions, known as CAPS (see above) (56). Although NLRP3 mutations are prototypically responsible for inflammasome activation, other mutations such as those involving IL-1 and IL-36 pathway genes may also induce inflammasome activation (13, 14). The symptoms of CAPS are the result of overexpression of IL-1β secondary to constitutive activation of the cytoplasmic macromolecular complex. Evaluation of patients with ND have also shown elevated serum and lesional tissues IL-β levels (50, 57, 58). In addition, keratinocytes exposed to ultraviolet B irradiation, contact allergens or in the setting of psoriasis activate similar inflammasome pathways resulting in IL-1β production and subsequent neutrophil localization and activation (59–61), although there is support from mouse models that bone marrow-derived cells with isolated NLRP3 mutations are sufficient to induce IL-1β associated cutaneous autoinflammation (62).

### Immune Signal Transduction

Immune cell proinflammatory signal transduction is inhibited by the tyrosine phosphatase known as Src homology region 2 (SH2) domain–containing phosphatase-1 (SHP-1) (63, 64). Dysfunctional activity of SHP-1 is associated with various diseases including multiple sclerosis, leukemia and psoriatic arthritis (65–69). SHP-1, also known as *PTPN6* (protein tyrosine phosphatase nonreceptor type 6), is encoded by the gene *Ptpn6*. Heterozygous mutations and splice variants of *Ptpn6* have been identified in patients with PG and SS (4).

Ptpn6^spin^ mice are the product of a Y208N (or Tyr208Asn) missense mutation leading to amino acid substitution in the carboxy-terminal SH2 domain of SHP-1. These mice develop severe cutaneous inflammation driven by overexpression of IL-1α (6). Histopathologically, the inflammatory cutaneous lesions resemble human NDs, with neutrophil-rich infiltrate and pustules within the epidermis, and are associated with neutrophilia. These data may support the role of mutations involving *Ptpn6* in triggering NDs in humans.

The Kanneganti group have utilized this murine model to characterize key regulatory components of neutrophil-mediated cutaneous autoinflammation. These authors have surprisingly shown that IL-1α signaling, but not IL-1β or caspase-1 associated inflammasome, plays a key role in orchestrating cutaneous autoinflammation in Ptpn6^spin^ mice (6). Their work has elucidated the complex regulation of the IL-1α pathway and identified a number of signaling components as pivotal in the development of cutaneous inflammation in Ptpn6^spin^ mice, such as IL-1 receptor (IL-1R), myeloid differentiation primary response gene 88 (MyD88) (7), spleen tyrosine kinase (Syk) (7), receptor interacting protein kinase 1 (RIPK1) (6), tumor growth factor-β activated kinase 1 (TAK1) (7) and apoptosis signal-regulating kinase 1 (ASK1) (8).

**Figure 1:**
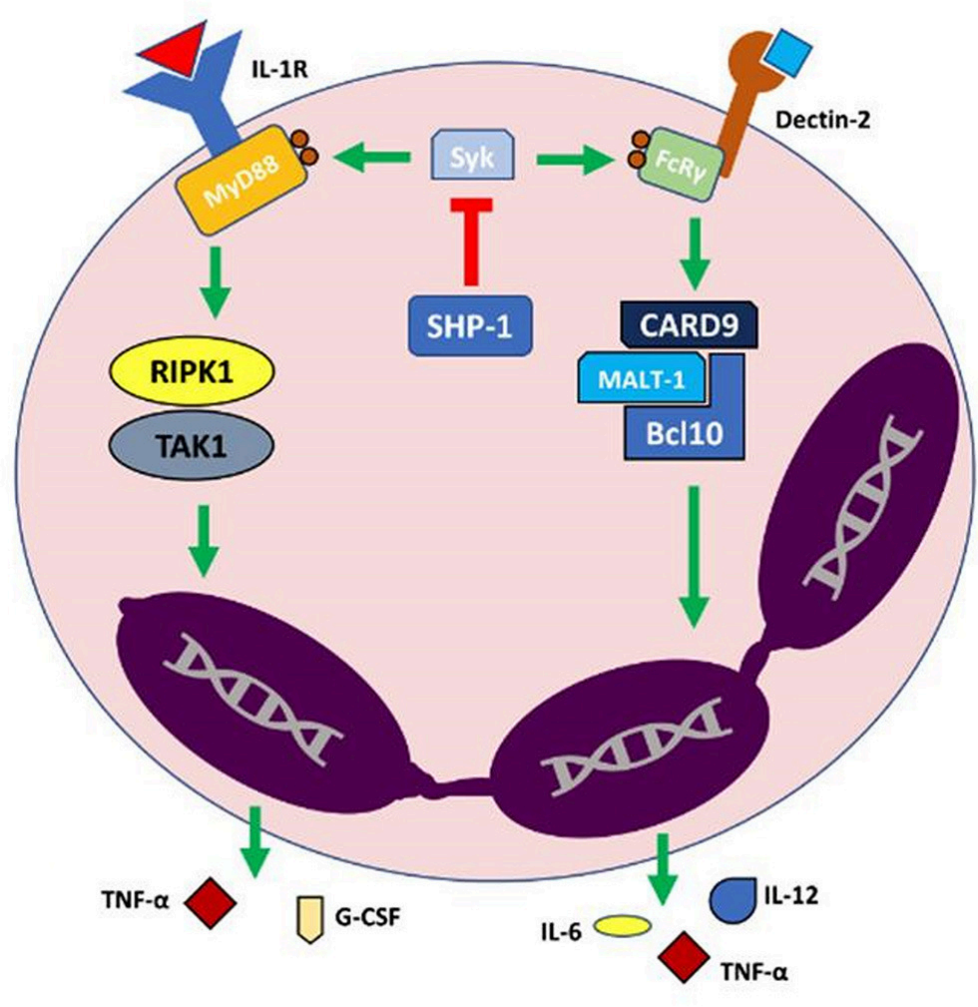
Model of IL-1α (Interleukin 1α)-mediated autoinflammation in neutrophilic dermatoses. In normal physiologic conditions SHP-1 (Src homology region 2 domain-containing phosphatase-1) acts to inhibit Syk (spleen tyrosine kinase) which subsequently downregulates the activation of IL-1R and Dectin-2 proinflammatory pathways. Dysfunction in SHP-1 leads to uncoupled Syk activity, subsequently leading to a neutrophil-driven autoinflammatory state via an exaggerated release of proinflammatory cytokines and other effector molecules. Bcl10, B-cell lymphoma/leukemia 10; CARD9, Caspase recruitment domain-containing protein 9; FcRy, Fc receptor common γ chain; G-CSF, Granulocyte Colony Stimulating Factor; IL-1R, Interleukin 1 receptor; MALT-1, Mucosa-associated lymphoid tissue lymphoma translocation protein 1; MyD88, Myeloid differentiation primary response 88; RIPK1, Receptor-interacting serine/threonine-protein kinase 1; TAK1, Transforming growth factor beta-activated kinase 1; TNF-α, Tumor necrosis factor α.

SHP-1 was also shown to regulate IL-1α signaling primarily through preventing phosphorylation of MyD88 by Syk (7). This pathway is illustrated in Figure 1. Of note, the Kanneganti group showed that inflammation in Ptpn6^spin^ mice is independent of toll-like receptors (TLRs), IFN-α/β receptor (IFNAR), integrin β-3 (ITGB3), NOD2–RIPK2 signaling, and independent of stimulator of IFN genes (STING) (7, 70). Support for unique role of IL-1α signaling in the development of cutaneous inflammation in Ptpn6^spin^ mice is strengthened by ruling out the influence of these additional innate immune signaling pathways (7, 70).

Recently, CARD9 signal transduction was identified as an essential mediator of cutaneous inflammation in Ptpn6^spin^ mice (71).

CARD9 is an adapter protein downstream of Syk and is central transducer for multiple innate signaling pathways including the C type lectin receptors, Dectin-1 and Mincle pathways (72). CARD9 forms a complex with MALT1 (mucosa-associated lymphoid tissue lymphoma translocation protein 1) and BCL10 (B-cell lymphoma/leukemia 10) leading to initiation of the NF-κB (nuclear factor kappa-light-chain-enhancer of activated B cells) signaling cascade and increased transcription of pro-inflammatory cytokines (TNF and IL-6) resulting in T helper (Th)17 polarization (73).

As previously discussed, IL-17 is a key signaling molecule in ND pathogenesis. CARD9 is thus linked to cytokine production, innate anti-fungal immunity, and myeloid cell activation (72). Identification of the key role of CARD9 in the autoinflammatory signaling pathway suggests the interconnection between NDs and other autoinflammatory conditions. Interestingly, CARD9 mutations are associated with IBD (74) and deletion in CARD9 significantly dampens the IL1-mediated cutaneous inflammatory disease in a mouse model knockout for CARD9 (70). Aberrant signaling involving SHP-1, Syk, and CARD9 can lead to neutrophil-mediated autoinflammation driven by overproduction of IL-1α. Thus, blocking these signals may provide a novel approach to design effective therapeutic strategies to treat NDs.

## Pathogenesis-Driven Treatment of Neutrophilic Dermatoses

Several lines of evidence indicate that IL-1 blockers, namely anakinra, rilonacept, gevokizumab, and canakinumab, are effective in the treatment of skin manifestations of different autoinflammatory disorders. The introduction of these pharmacological agents represents a breakthrough not only in the management of monogenic inherited autoinflammatory diseases but also of skin diseases where neutrophils play a crucial pathogenic role. More specifically, Brenner et al. (75) reported complete healing of PG and acne in a patient affected by PAPA after 1 month-therapy with anakinra 100 mg/day. Similarly, canakinumab 150 mg every 8 weeks led to complete remission of PG and acne lesions in another PAPA patient. Two case reports are present in the literature on two patients with PASH treated with anakinra, who both experienced a partial remission of skin lesions (11, 76). In an open-label, proof of concept study conducted on six patients with active PG who were given three subcutaneous injections once every 4 weeks of the anti-IL-1β monoclonal antibody gevokizumab, complete remission of PG was observed in four patients, a partial response in one patient and another one did not respond. (ClinicalTrials.gov Identifier: NCT01882504) Conversely, a phase 3 trial demonstrated limited effectiveness of the same drug on PG patients. (ClinicalTrials.gov Identifier: NCT02326740 and NCT02315417) Complete PG clearance under canakinumab treatment has been described in two patients after 3 (77) and 12 months (78), respectively. Canakinumab effectiveness has also been assessed in an open-label study on five PG patients unresponsive to systemic steroids, with three out of five achieving complete remission at 16-week follow-up visit (79). A prompt and long-lasting response to anakinra in terms of both cutaneous and systemic manifestations has been described in a patient with SS (80). In another SS patient, Kluger et al. observed that, despite a relatively rapid response to anakinra, both skin and systemic symptoms relapsed upon drug withdrawal (81). Amazan et al. reported on a woman with steroid- and anti-TNFα- refractory APF who experienced complete healing of her lesions on a regimen of 100 mg/day anakinra (82).

A rapid and robust clinical response to secukinumab, an anti-IL-17 monoclonal antibody, was reported in an adolescent with severe cutaneous manifestations due to DITRA (83). In addition to the latter clinical observation, a pathomechanistic link between IL-36 and Th17 differentiation may be postulated also based on the findings by Carrier et al. (26), who showed a direct correlation between IL-36 gene expression and IL-17 levels in the lesional skin of psoriatic patients.

Future perspectives in the management of PG involve the IL-1α blockade. IL-1α overproduction has been demonstrated in response to deregulated SHP-1 activity triggering a severe neutrophil-mediated inflammatory disease that develops independently of inflammasome. Based on these findings, there is an ongoing phase 2 open-label trial using bermekimab, an IL-1α inhibitor, in PG (ClinicalTrials.gov Identifier: NCT01965613).

## Conclusions

NDs are a complex, variable and heterogenous group of diseases which have significant overlap in presentation and pathogenesis. Our understanding of the molecular mechanisms of NDs is based primarily on the discovery of familial variants, classification of autoinflammatory syndromes and development of mouse models. While understanding of disease mechanisms has not yet been completely elucidated, NDs are recognized as a polygenic multifactorial disease process which involves dysfunctional cellular signaling mediated by pathways mainly related to inflammasome and IL-1 with the contributory role of IL-17 and other effector molecules. The precise elucidation of the above-mentioned pathologic mechanisms will pave the way to tailored treatments for patients with different NDs.

## Author Contributions

AO-L, MC, and AM designed and reviewed the paper and contributed in drafting the manuscript. MH drafted the manuscript. GG and DM contributed in drafting and reviewing the manuscript. All the authors approved the final version of the manuscript.

### Conflict of Interest Statement

The authors declare that the research was conducted in the absence of any commercial or financial relationships that could be construed as a potential conflict of interest.

## References

[1] Marzano AV Ceccherini I Gattorno M Fanoni D Caroli F Rusmini M. Association of pyoderma gangrenosum, acne, and suppurative hidradenitis (PASH) shares genetic and cytokine profiles with other autoinflammatory diseases. Medicine. (2014) 93:e187. 10.1097/MD.0000000000000187 25501066 PMC4602806

[2] Marzano AV Damiani G Ceccherini I Berti E Gattorno M Cugno M. Autoinflammation in pyoderma gangrenosum and its syndromic form (pyoderma gangrenosum, acne and suppurative hidradenitis). Br J Dermatol. (2017) 176:1588–98. 10.1111/bjd.15226 27943240

[3] Newman B Cescon D Domenchini A Siminovitch KA CD2BP1 and CARD15 mutations are not associated with pyoderma gangrenosum in patients with inflammatory bowel disease. J Invest Dermatol. (2004) 122:1054–6. 10.1111/j.0022-202X.2004.22430.x 15102098

[4] Nesterovitch AB Gyorfy Z Hoffman MD Moore EC Elbuluk N Tryniszewska B. Alteration in the gene encoding protein tyrosine phosphatase nonreceptor type 6 (PTPN6/SHP1) may contribute to neutrophilic dermatoses. Am J Pathol. (2011) 178:1434–41. 10.1016/j.ajpath.2010.12.035 21406173 PMC3078441

[5] Guenova E Teske A Fehrenbacher B Hoerber S Adamczyk A Schaller M. Interleukin 23 expression in pyoderma gangrenosum and targeted therapy with ustekinumab. Arch Dermatol. (2011) 147:1203–5. 10.1001/archdermatol.2011.168 21680759

[6] Lukens JR Vogel P Johnson GR Kelliher MA Iwakura Y Lamkanfi M. RIP1-driven autoinflammation targets IL-1alpha independently of inflammasomes and RIP3. Nature. (2013) 498:224–7. 10.1038/nature12174 23708968 PMC3683390

[7] Gurung P Fan G Lukens JR Vogel P Tonks NK Kanneganti TD. Tyrosine kinase SYK licenses MyD88 adaptor protein to instigate IL-1alpha-mediated inflammatory disease. Immunity. (2017) 46:635–48. 10.1016/j.immuni.2017.03.014 28410990 PMC5501252

[8] Tartey S Gurung P Dasari TK Burton A Kanneganti TD. ASK1/2 signaling promotes inflammation in a mouse model of neutrophilic dermatosis. J Clin Invest. (2018) 128:2042–7. 10.1172/JCI98446 29629899 PMC5919885

[9] Demidowich AP Freeman AF Kuhns DB Aksentijevich I Gallin JI Turner ML. Brief report: genotype, phenotype, and clinical course in five patients with PAPA syndrome (pyogenic sterile arthritis, pyoderma gangrenosum, and acne). Arthritis Rheum. (2012) 64:2022–7. 10.1002/art.34332 22161697 PMC3737487

[10] Wise CA Gillum JD Seidman CE Lindor NM Veile R Bashiardes S. Mutations in CD2BP1 disrupt binding to PTP PEST and are responsible for PAPA syndrome, an autoinflammatory disorder. Hum Mol Gen. (2002) 11:961–9. 10.1093/hmg/11.8.961 11971877

[11] Braun-Falco M Kovnerystyy O Lohse P Ruzicka T. Pyoderma gangrenosum, acne, and suppurative hidradenitis (PASH)–a new autoinflammatory syndrome distinct from PAPA syndrome. J Am Acad Dermatol. (2012) 66:409–15. 10.1016/j.jaad.2010.12.025 21745697

[12] Marzano AV Trevisan V Gattorno M Ceccherini I De Simone C Crosti C. Pyogenic arthritis, pyoderma gangrenosum, acne, and hidradenitis suppurativa (PAPASH): a new autoinflammatory syndrome associated with a novel mutation of the PSTPIP1 gene. JAMA Dermatol. (2013) 149:762–4. 10.1001/jamadermatol.2013.2907 23571383

[13] Aksentijevich I Masters SL Ferguson PJ Dancey P Frenkel J van Royen-Kerkhoff A. An autoinflammatory disease with deficiency of the interleukin-1-receptor antagonist. N Engl J Med. (2009) 360:2426–37. 10.1056/NEJMoa0807865 19494218 PMC2876877

[14] Marrakchi S Guigue P Renshaw BR Puel A Pei XY Fraitag S. Interleukin-36 receptor antagonist deficiency and generalized pustular psoriasis. N Engl J Med. (2011) 365:620–8. 10.1056/NEJMoa1013068 21848462

[15] Onoufriadis A Simpson MA Pink AE Di Meglio P Smith CH Pullabhatla V. Mutations in IL36RN/IL1F5 are associated with the severe episodic inflammatory skin disease known as generalized pustular psoriasis. Am J Hum Genet. (2011) 89:432–7. 10.1016/j.ajhg.2011.07.022 21839423 PMC3169817

[16] Kastner DL Aksentijevich I Goldbach-Mansky R. Autoinflammatory disease reloaded: a clinical perspective. Cell. (2010) 140:784–90. 10.1016/j.cell.2010.03.002 20303869 PMC3541025

[17] Marzano AV Damiani G Genovese G Gattorno M. A dermatologic perspective on autoinflammatory diseases. Clin Exp Rheumatol. (2018) 36 (Suppl 110):32–8. 29742056

[18] Marzano AV Borghi A Wallach D Cugno M. A comprehensive review of neutrophilic diseases. Clin Rev Allergy Immunol. (2018) 54:114–30. 10.1007/s12016-017-8621-8 28688013

[19] McDermott MF Aksentijevich I Galon J McDermott EM Ogunkolade BW Centola M. Germline mutations in the extracellular domains of the 55 kDa TNF receptor, TNFR1, define a family of dominantly inherited autoinflammatory syndromes. Cell. (1999) 97:133–44. 10.1016/S0092-8674(00)80721-7 10199409

[20] Satoh TK Mellett M Contassot E French LE. Are neutrophilic dermatoses autoinflammatory disorders? Br J Dermatol. (2018) 178:603–13. 10.1111/bjd.15105 27905098

[21] Prat L Bouaziz JD Wallach D Vignon-Pennamen MD Bagot M. Neutrophilic dermatoses as systemic diseases. Clin Dermatol. (2014) 32:376–88. 10.1016/j.clindermatol.2013.11.004 24767185

[22] Beer HD Contassot E French LE. The inflammasomes in autoinflammatory diseases with skin involvement. J Invest Dermatol. (2014) 134:1805–10. 10.1038/jid.2014.76 24599175

[23] Reddy S Jia S Geoffrey R Lorier R Suchi M Broeckel U. An autoinflammatory disease due to homozygous deletion of the IL1RN locus. N Engl J Med. (2009) 360:2438–44. 10.1056/NEJMoa0809568 19494219 PMC2803085

[24] Blumberg H Dinh H Trueblood ES Pretorius J Kugler D Weng N. Opposing activities of two novel members of the IL-1 ligand family regulate skin inflammation. J Exp Med. (2007) 204:2603–14. 10.1084/jem.20070157 17908936 PMC2118475

[25] Bassoy EY Towne JE Gabay C. Regulation and function of interleukin-36 cytokines. Immunol Rev. (2018) 281:169–78. 10.1111/imr.12610 29247994

[26] Carrier Y Ma H-L Ramon HE Napierata L Small C O'Toole M. Inter-regulation of Th17 cytokines and the IL-36 cytokines *in vitro* and *in vivo*: implications in psoriasis pathogenesis. J Invest Dermatol. (2011) 131:2428–37. 10.1038/jid.2011.234 21881584

[27] Milora KA Fu H Dubaz O Jensen LE Unprocessed interleukin-36α regulates psoriasis-like skin inflammation in co-operation with interleukin-1. J Invest Dermatol. (2015) 135:2992–3000. 10.1038/jid.2015.289 26203636 PMC4648684

[28] Johnston A Xing X Wolterink L Barnes DH Yin Z Reingold L. IL-1 and IL-36 are dominant cytokines in generalized pustular psoriasis. J Allergy Clin Immunol. (2017)140:109–20. 10.1016/j.jaci.2016.08.056 28043870 PMC5494022

[29] Shoham NG Centola M Mansfield E Hull KM Wood G Wise CA. Pyrin binds the PSTPIP1/CD2BP1 protein, defining familial Mediterranean fever and PAPA syndrome as disorders in the same pathway. Proc Natl Acad Sci USA. (2003) 100:13501–6. 10.1073/pnas.2135380100 14595024 PMC263843

[30] Navarini AA Satoh TK French LE. Neutrophilic dermatoses and autoinflammatory diseases with skin involvement–innate immune disorders. Semin Immunopathol. (2016) 38:45–56. 10.1007/s00281-015-0549-6 26620372

[31] al-Rimawi HS Abuekteish FM Daoud AS Oboosi MM. Familial pyoderma gangrenosum presenting in infancy. Eur J Pediatr. (1996) 155:759–62. 10.1007/s004310050482 8874107

[32] Khandpur S Mehta S Reddy BS. Pyoderma gangrenosum in two siblings: a familial predisposition. Pediatr Dermatol. (2001) 18:308–12. 10.1046/j.1525-1470.2001.01936.x 11576404

[33] Masters SL Lagou V Jeru I Baker PJ Van Eyck L Parry DA. Familial autoinflammation with neutrophilic dermatosis reveals a regulatory mechanism of pyrin activation. Sci Transl Med. (2016) 8:332ra345. 10.1126/scitranslmed.aaf1471 27030597

[34] Yorimitsu T Nair U Yang Z Klionsky DJ. Endoplasmic reticulum stress triggers autophagy. J Biol Chem. (2006) 281:30299–304. 10.1074/jbc.M607007200 16901900 PMC1828866

[35] Sidiq T Yoshihama S Downs I Kobayashi KS. Nod2: a critical regulator of ileal microbiota and Crohn's disease. Front Immunol. (2016) 7:367. 10.3389/fimmu.2016.00367 27703457 PMC5028879

[36] Nakahira K Haspel JA Rathinam VA Lee SJ Dolinay T Lam HC. Autophagy proteins regulate innate immune responses by inhibiting the release of mitochondrial DNA mediated by the NALP3 inflammasome. Nat Immunol. (2011) 12:222–30. 10.1038/ni.1980 21151103 PMC3079381

[37] Zhou R Yazdi AS Menu P Tschopp J. A role for mitochondria in NLRP3 inflammasome activation. Nature. (2011) 469:221–5. 10.1038/nature09663 21124315

[38] Harris J Hartman M Roche C Zeng SG O'Shea A Sharp FA. Autophagy controls IL-1beta secretion by targeting pro-IL-1beta for degradation. J Biol Chem. (2011) 286:9587–97. 10.1074/jbc.M110.202911 21228274 PMC3058966

[39] Soehnlein O Steffens S Hidalgo A Weber C. Neutrophils as protagonists and targets in chronic inflammation. Nat Rev Immunol. (2017) 17:248–61. 10.1038/nri.2017.10 28287106

[40] von Vietinghoff S Ley K. Homeostatic regulation of blood neutrophil counts. J Immunol. (2008) 181:5183–8. 10.4049/jimmunol.181.8.5183 18832668 PMC2745132

[41] Stark MA Huo Y Burcin TL Morris MA Olson TS Ley K. Phagocytosis of apoptotic neutrophils regulates granulopoiesis via IL-23 and IL-17. Immunity. (2005) 22:285–94. 10.1016/j.immuni.2005.01.011 15780986

[42] von Vietinghoff S Ley K. IL-17A controls IL-17F production and maintains blood neutrophil counts in mice. J Immunol. (2009) 183:865–73. 10.4049/jimmunol.0804080 19542376 PMC2759196

[43] Kawakami T Ohashi S Kawa Y Takahama H Ito M Soma Y. Elevated serum granulocyte colony-stimulating factor levels in patients with active phase of sweet syndrome and patients with active behcet disease: implication in neutrophil apoptosis dysfunction. Arch Dermatol. (2004) 140:570–4. 10.1001/archderm.140.5.570 15148101

[44] Uhara H Saida T Nakazawa H Ito T. Neutrophilic dermatoses with acute myeloid leukemia associated with an increase of serum colony-stimulating factor. J Am Acad Dermatol. (2008) 59:S10–12. 10.1016/j.jaad.2007.08.026 18625369

[45] Reuss-Borst MA Pawelec G Saal JG Horny HP Muller CA Waller HD. Sweet's syndrome associated with myelodysplasia: possible role of cytokines in the pathogenesis of the disease. Br J Hematol. (1993) 84:356–8. 10.1111/j.1365-2141.1993.tb03083.x 7691149

[46] Hattori H Hoshida S Yoneda S Sweet's syndrome associated with recurrent fever in a patient with trisomy 8 myelodysplastic syndrome. Int J Hematol. (2003) 7:383–6. 10.1007/BF02982648 12774928

[47] Prevost-Blank PL Shwayder TA. Sweet's syndrome secondary to granulocyte colony-stimulating factor. J Am Acad Dermatol. (1996) 35:995–7. 10.1016/S0190-9622(96)90132-2 8959967

[48] Marzano AV Cugno M Trevisan V Fanoni D Venegoni L Berti E. Role of inflammatory cells, cytokines and matrix metalloproteinases in neutrophil-mediated skin diseases. Clin Exp Immunol. (2010) 162:100–7. 10.1111/j.1365-2249.2010.04201.x 20636397 PMC2990935

[49] Marzano AV Cugno M Trevisan V Lazzari R Fanoni D Berti E. Inflammatory cells, cytokines and matrix metalloproteinases in amicrobial pustulosis of the folds and other neutrophilic dermatoses. Int J Immunopathol Immunopharmacol. (2011) 24:451–60. 10.1177/039463201102400218 21658319

[50] Marzano AV Fanoni D Antiga E Quaglino P Caproni M Crosti C. Expression of cytokines, chemokines and other effector molecules in two prototypic autoinflammatory skin diseases, pyoderma gangrenosum and Sweet's syndrome. Clin Exp Immunol. (2014) 178:48–56. 10.1111/cei.12394 24903614 PMC4360193

[51] Marzano AV Tavecchio S Berti E Gelmetti C Cugno M. Cytokine and chemokine profile in amicrobial pustulosis of the folds: evidence for autoinflammation. Medicine. (2015) 94:e230. 10.1097/MD.0000000000002301 26683967 PMC5058939

[52] Marzano AV Tavecchio S Berti E Gelmetti C Cugno M. Paradoxical autoinflammatory skin reaction to tumor necrosis factor alpha blockers manifesting as amicrobial pustulosis of the folds in patients with inflammatory bowel diseases. Medicine. (2015) 94:e1818. 10.1097/MD.0000000000001818 26559252 PMC4912246

[53] Oka M Berking C Nesbit M Satyamoorthy K Schaider H Murphy G. Interleukin-8 overexpression is present in pyoderma gangrenosum ulcers and leads to ulcer formation in human skin xenografts. Lab Invest. (2000) 80:595–604. 10.1038/labinvest.3780064 10780675

[54] Cataisson C Pearson AJ Torgerson S Nedospasov SA Yuspa SH. Protein kinase C alpha-mediated chemotaxis of neutrophils requires NF-kappa B activity but is independent of TNF alpha signaling in mouse skin *in vivo*. J Immunol. (2005) 174:1686–92. 10.4049/jimmunol.174.3.1686 15661932

[55] de Torre-Minguela C Mesa Del Castillo P Pelegrin P The NLRP3 and pyrin inflammasomes: implications in the pathophysiology of autoinflammatory diseases. Front Immunol. (2017) 8:43 10.3389/fimmu.2017.00043 28191008 PMC5271383

[56] Hoffman HM Wanderer AA Broide DH. Familial cold autoinflammatory syndrome: phenotype and genotype of an autosomal dominant periodic fever. J Allergy Clin Immunol. (2001) 108:615–20. 10.1067/mai.2001.118790 11590390 PMC4321996

[57] Imhof L Meier B Frei P Kamarachev J Rogler G Kolios A. Severe sweet's syndrome with elevated cutaneous interleukin-1beta after azathioprine exposure: case report and review of the literature. Dermatology. (2015) 230:293–8. 10.1159/000371879 25791317

[58] Giasuddin AS El-Orfi AH Ziu MM El-Barnawi NY. Sweet's syndrome: is the pathogenesis mediated by helper T cell type 1 cytokines? J Am Acad Dermatol. (1998) 39:940–3. 10.1016/S0190-9622(98)70266-X 9843005

[59] Dombrowski Y Peric M Koglin S Kammerbauer C Göss C Anz D. Cytosolic DNA triggers inflammasome activation in keratinocytes in psoriatic lesions. Sci Transl Med. (2011) 3:82ra38. 10.1126/scitranslmed.3002001 21562230 PMC3235683

[60] Feldmeyer L Keller M Niklaus G Hohl D Werner S Beer HD. The inflammasome mediates UVB-induced activation and secretion of interleukin-1beta by keratinocytes. Curr Biol. (2007) 17:1140–5. 10.1016/j.cub.2007.05.074 17600714

[61] Watanabe H Gaide O Petrilli V Martinon F Contassot E Roques S. Activation of the IL-1beta-processing inflammasome is involved in contact hypersensitivity. J Invest Dermatol. (2007) 127:1956–63. 10.1038/sj.jid.5700819 17429439

[62] Meng G Zhang F Fuss I Kitani A Strober W. A mutation in the Nlrp3 gene causing inflammasome hyperactivation potentiates Th17 cell-dominant immune responses. Immunity. (2009) 30:860–74. 10.1016/j.immuni.2009.04.012 19501001 PMC2764254

[63] Lukens JR Kanneganti TD. SHP-1 and IL-1alpha conspire to provoke neutrophilic dermatoses. Rare Dis. (2014) 2:e27742. 10.4161/rdis.27742 25054090 PMC4091500

[64] Zhang J Somani AK Siminovitch KA. Roles of the SHP-1 tyrosine phosphatase in the negative regulation of cell signalling. Semin Immunol. (2000) 12:361–78. 10.1006/smim.2000.0223 10995583

[65] Prestin K Olbert M Hussner J Volzke H Meyer Zu Schwabedissen HE. Functional assessment of genetic variants located in the promoter of SHP1 (NR0B2). Pharmacogenet Genomics. (2017) 27:410–5. 10.1097/FPC.0000000000000310 28873070

[66] Cao H Hegele RA. Identification of polymorphisms in the human SHP1 gene. J Hum Gen. (2002) 47:445–7. 10.1007/s100380200052 12181644

[67] Christophi GP Hudson CA Gruber RC Christophi RL Gruber RC Mersich AT. SHP-1 deficiency and increased inflammatory gene expression in PBMCs of multiple sclerosis patients. Lab Invest. (2008) 88:243–55. 10.1038/labinvest.3700720 18209728 PMC2883308

[68] Eriksen KW Woetmann A Skov L Krejsgaard T Bovin LF Hansen ML. Deficient SOCS3 and SHP-1 expression in psoriatic T cells. J Invest Dermatol. (2010) 130:1590–7. 10.1038/jid.2010.6 20130595

[69] Tibaldi E Brunati AM Zonta F Frezzato F Gattazzo C Zambello R. Lyn-mediated SHP-1 recruitment to CD5 contributes to resistance to apoptosis of B-cell chronic lymphocytic leukemia cells. Leukemia. (2011) 25:1768–81. 10.1038/leu.2011.152 21701493

[70] You RI Chu CL SHP-1 (PTPN6) keeps the inflammation at bay: limiting IL-1alpha-mediated neutrophilic dermatoses by preventing Syk kinase activation. Cell Mol Immunol. (2017) 14:881–3. 10.1038/cmi.2017.59 PMC5675957 28690326

[71] Tartey S Gurung P Samir P Burton A Kanneganti TD. Cutting edge: dysregulated CARD9 signaling in neutrophils drives inflammation in a mouse model of neutrophilic dermatoses. J Immunol. (2018) 201:1639–44. 10.4049/jimmunol.1800760 30082320 PMC6483079

[72] Gross O Gewies A Finger K Schäfer M Sparwasser T Peschel C. Card9 controls a non-TLR signalling pathway for innate anti-fungal immunity. Nature. (2006) 442:651–6. 10.1038/nature04926 16862125

[73] Bertin J Guo Y Wang L Srinivasula SM Jacobson MD Poyet JL. CARD9 is a novel caspase recruitment domain-containing protein that interacts with BCL10/CLAP and activates NF-kappa B. J Biol Chem. (2000) 275:41082–6. 10.1074/jbc.C000726200 11053425

[74] Leshchiner ES Rush JS Durney MA Cao Z Dančík V Chittick B. Small-molecule inhibitors directly target CARD9 and mimic its protective variant in inflammatory bowel disease. Proc Natl Acad Sci USA. (2017) 114:11392–7. 10.1073/pnas.1705748114 29073062 PMC5664502

[75] Brenner M Ruzicka T Plewig G Thomas P Herzer P. Targeted treatment of pyoderma gangrenosum in PAPA (pyogenic arthritis, pyoderma gangrenosum and acne) syndrome with the recombinant human interleukin-1 receptor antagonist anakinra. Br J Dermatol. (2009) 161:1199–201. 10.1111/j.1365-2133.2009.09404.x 19673875

[76] Jennings L Molloy O Quinlan C Kelly G O'Kane M. Treatment of pyoderma gangrenosum, acne, suppurative hidradenitis (PASH) with weight-based anakinra dosing in a Hepatitis B carrier. Int J Dermatol. (2017) 56:e128–e129. 10.1111/ijd.13528 28239847

[77] Galimberti RL Vacas AS Bollea Garlatti ML Torre AC. The role of interleukin-1β in pyoderma gangrenosum. JAAD Case Rep. (2016) 2:366–8. 10.1016/j.jdcr.2016.07.007 27709123 PMC5043387

[78] Jaeger T Andres C Grosber M Zirbs M Hein R Ring J. Pyoderma gangrenosum and concomitant hidradenitis suppurativa–rapid response to canakinumab (anti-IL-1β). Eur J Dermatol. (2013) 23:408–10. 10.1684/ejd.2013.2018. 23816498

[79] Kolios AG Maul JT Meier B Kerl K Traidl-Hoffmann C Hertl M. Canakinumab in adults with steroid-refractory pyoderma gangrenosum. Br J Dermatol. (2015) 173:1216–23. 10.1111/bjd.14037 26471257

[80] Delluc A Limal N Puéchal X Francès C Piette JC Cacoub P. Efficacy of anakinra, an IL1 receptor antagonist, in refractory Sweet syndrome. Ann Rheum Dis. (2008) 67:278–9. 10.1136/ard.2006.068254 18192308

[81] Kluger N Gil-Bistes D Guillot B Bessis D. Efficacy of anti-interleukin-1 receptor antagonist anakinra (Kineret®) in a case of refractory Sweet's syndrome. Dermatology. (2011) 222:123–7. 10.1159/000326112 21464561

[82] Amazan E Ezzedine K Mossalayi MD Taieb A Boniface K Seneschal J. Expression of interleukin-1 alpha in amicrobial pustulosis of the skin folds with complete response to anakinra. J Am Acad Dermatol. (2014) 71:e53–6. 10.1016/j.jaad.2013.12.041 25037815

[83] Cordoro KM Ucmak D Hitraya-Low M Rosenblum MD Liao W. Response to interleukin (IL)-17 inhibition in an adolescent with severe manifestations of IL-36 receptor antagonist deficiency (DITRA). JAMA Dermatol. (2017) 153:106–8. 10.1001/jamadermatol.2016.3490 27760239

